# Women in ASHE: finding fulfillment via the road less traveled

**DOI:** 10.1017/ash.2023.431

**Published:** 2023-10-23

**Authors:** Kelly Cawcutt

**Affiliations:** Divisions of Infectious Diseases and Pulmonary & Critical Care Medicine, University of Nebraska Medical Center, Omaha, NE, USA

**1. Tell us about your nontraditional career path in infection prevention (IP) and hospital epidemiology. How has working as an intensivist has changed your approach to IP? What lessons from the ICU setting can be applied to the rest of the hospital in terms of HAI prevention?**

My career in medicine truly started in my childhood. I had multiple health issues prompting more visits to the doctor than I can remember. I do recall always being intrigued by the posters of human anatomy on the walls and asking questions about those posters as it related to myself and my health. It wasn’t until I was much older that I realized not every kid had multiple physicians or had undergone surgery. As I wrapped my head around that reality, I knew I loved two things—science and education. Early moments of curiosity, realization, and feedback helped plant the seed—at first, I seriously considered education as a career, but a high school teacher and mentor persuaded me to pursue medicine. As a medical student, my beloved grandfather died in my training hospital ICU over Christmas of septic shock secondary to a MRSA bloodstream infection. In retrospect, this experience was a significant part of my drive to pursue a unified career path in critical care medicine (CCM), infectious diseases (IDs) and IP, although I did not recognize it until years later.

As an internal medicine resident, I quickly found that I loved the complexity and acuity of the ICU. During my first ID rotation, I noted significant overlap between these subspecialties. The prospect of combining ID and critical care was incredibly compelling as both require vast medical knowledge of multiple diseases impacting any organ system, at any time, in various ways. ICU patients either arrived infected or were at heightened risk of hospital-onset infections amidst the array of invasive devices. Moreover, recognition of the frequent need for antimicrobials and the continuous diagnostic uncertainty in the critically ill population was a lightbulb moment helping to shape my career path.

However, becoming an ID and critical care (ID/CCM) physician was far more complex than I expected. I wanted to remain in academic medicine but lacked a mentor with combined training, and at the time, dual fellowship tracks were scarce. I became my own advocate and emailed programs around the country asking if 1) they would consider supporting a combined ID/CCM fellowship option, and 2) if they would consider hiring an ID/CCM physician.

Suffice it to say, I received a lot of “nos” and many responses stating that I would need to be more competitive than other pulmonary/critical care candidates to justify the effort allocation and meet financial benchmarks. Mayo Clinic in Rochester, MN, provided the “yes,” I desired; and I completed my CCM and ID fellowships there, along with a Masters in Clinical and Translational Science.

They say, “never waste a good crisis,” and in my career, Ebola was the first crisis. I was looking for faculty positions during the Ebola outbreak, which strengthened my pitch for the niche value of an ID/CCM trained physician. I had the opportunity to meet Dr. Angela Hewlett from the University of Nebraska Medical Center at the Remington ID course in Utah. She spoke passionately about her experiences in the biocontainment unit working with Ebola patients, and the need for collaboration between ID and CCM physicians. UNMC struck me as an organization that truly recognized the value of a dually trained ID/CCM physician. I joined the faculty at UNMC in January 2016 where I currently serve as Associate Professor in ID and CCM (with active clinical effort in both services), Associate Director of Infection Control, Director of Medical Quality, and Co-Director of Digital Innovation and Social Media Strategy for the ID division. In addition, I have served as an active member of the biocontainment unit team.

I find that being a CCM and ID physician allows me to relate better to clinical staff throughout the hospital and engage with teams through my work in Quality and Infection Control. These experiences provide a broader context for clinical care, and this knowledge helps me liaise between historically siloed specialties in academic medicine, build rapport, and help create meaningful, impactful change for our patients and the healthcare workforce overall.

I often state that as an ID doctor, sometimes I practice CCM, but as an intensivist, I practice ID every day with every patient. Each ICU patient has a suspected or known infection or is at increased risk of developing an infection due to their acuity of illness, invasive devices, and the heavy antibiotic exposure. As an IP director, I’m uniquely served by a deep understanding of the implications of HAIs such as ventilator-associated events/pneumonias (VAE/VAPs) or central line-associated bloodstream infections and best practices for HAI prevention. As an intensivist, serving on the frontline performing and supervising insertion and maintenance procedures provides much needed clinical context and social capital to help implement change. These phenomena were acutely put into practice during the COVID pandemic, which required much from all healthcare workers, particularly those in CCM, ID, and IP.

**2. What are the biggest internal drivers of your success?**

First, inherently, I have always been driven to succeed, which is a drive that is supported and celebrated by my family and friends. I was driven to medicine as personal experiences and scientific interests combined with a vision to help others. The appeal of greater impact beyond the individual patient also draws me to extramural work within medical societies such as ABIM, CHEST, IDSA, SCCM, SHEA, and ACP. Finally, although still relatively early in my career, I think a lot about legacy. I am honored to be a first-generation college graduate and first physician in my family. Inspiring and supporting others in health care is another means toward a lasting impact. I deeply believe that we need to develop individuals and provide equitable opportunities in medicine. Finally, I must admit that I’m driven to prove wrong all who insisted that a combined academic career in CCM, ID, and IP was impossible.

**3. Describe a pivotal mentor relationship that altered the trajectory of your career**

First was Dr. Kianoush Kashani, who really was the first physician to whole-heartedly believe in me and my career vision. He provided me with the opportunities and support that jumpstarted my career.

Dr. Mark Rupp, my division chief, has been a mentor and sponsor since our first meeting. He saw the potential within a combined career path advocated for me to continue to both work and develop in both specialties. He has sponsored my administrative roles within Infection Prevention and Quality and nominated me for the opportunity to pen this piece in ASHE. I certainly would not have become this person without his influence and guidance.

Finally, Dr. Andre Kalil, has served as a research and career mentor, a sponsor, and a kindred spirit in the ID and CCM world who has helped me navigate clinical and professional challenges in both spaces.

**4. How has taking on mentorship of others made you better as a healthcare leader?**

Mentorship, coaching, and sponsorship have been of paramount importance to my personal and professional development. I believe that at all career stages, we have something to offer others. An effective healthcare leader understands that everyone has experience, expertise, and ideas to offer, and we need to listen. Seeing mentees succeed provides profound joy, and on the hardest days, these mentoring moments remind me of the beauty and diversity of humanity. For me, mentoring helps mitigate burnout. My mentees are always teaching me, making me a better human, physician, and leader.

**5. What were some notable barriers in your career and how did you address them?**

All too often we read personal career stories which seem effortless and rarely have access to the deep, dark struggles. Certainly, I struggled as woman in a male-dominated specialty of CCM, with gender biases and a nontraditional career to defend. I was told repeatedly that I would not be recognized as an expert in both fields and was subject to frankly sexist comments about the sustainability of my professional choices as a wife and mother.

Other hurdles included completing my Masters, “learning the ropes” in Infection Control largely on the job, and finding opportunities to rise in leadership (internally and externally). This is an enduring challenge for those of us who straddle different faculty appointments, divisions, and medical societies.

At times I felt I had to exceed the qualifications of others or often felt pulled in different directions, which can be lonely and discouraging. Pandemic-related burnout amplified these issues and required me to focus more on myself.

A few key strategies I lean on include: 1) developing my circle of support for advice, strength, and courage; 2) self-sponsoring for opportunities and having my “elevator pitch” ready for selling my unique value and why I was seeking an opportunity; 3) seeking out formal training and skills in order to overcome barriers (which may require specifically asking for additional feedback to determine what these are), 4) addressing my own burnout, episodes of imposter phenomenon and developing stronger boundaries to ensure I maintained the mental and physical well-being I needed.

**6. You’re well known for your social medial presence and expertise on the use of these platforms to promote the science of stewardship, IP, and medicine. How has social media shaped your career in both positive and less positive ways? How has your relationship with SM changed over time (perhaps influenced by the pandemic)? Finally, what are 2–3 best practices you’d recommend for our readers?**

Social media provided an avenue to network, to develop relationships with others outside of my geographic area and has led to varied opportunities in my career. My professional engagement accelerated within the area of gender equity and related to ID. My overall view is that social media has a positive impact on careers, but there are certainly negative aspects and risks, which have increased over the past few years. I’ve had threats and been trolled, but nothing that has required a shutdown of my accounts. Social media also can have a negative mental health impact with scrolling through commentary, comparisons, mis- and dis-information, and inflammatory content. I also believe there are different seasons in all our lives in which our engagement via any conduit of communication shifts. My top three tips for managing social media include:

1. Consider the reason for engaging in social media—both the “why” and the “who” and cater content to both. For example, the goal is professional growth, sharing content and engaging in conversations with colleagues within your areas of expertise.
2. Be authentic and remember that these are platforms where content is shared very quickly and effectively. If you would not say it in a mic at a national meeting or show that photo during a large presentation, pause and assess why you are considering posting it.
3. Understand that it is ok to take a break when you need it; your health and sanity are far more important than trending topics or the comments on a post.

**7. What career advice would you share with young professionals just starting out in epidemiology, public health, or stewardship?**

Epidemiology, public health, and stewardship often feel intimidating as in-depth training may not be provided until you accept a role—“learning on the job” is completely acceptable! Step into the discomfort—that is where we find the greatest growth and potential. Do not be afraid not have the answers and know when to ask for help. Remember—health care requires multidisciplinary teams that work well together. Invest in building the teams and relationships you require to be successful in your role(s) but remember to invest in your own growth as well—build your team of mentors, sponsors, peer-coaches, and supporters! Do not be afraid of unconventional routes, trying innovative ideas, or occasionally, failing. Engage with professional societies, such as SHEA to learn, find mentors, and build collaborative networks. Finally, find a peer group to rely on when you have questions and to bond with at conferences.

**8. Finally, what nonmedical book, essay, or podcast is an absolute “must consume,” in your opinion?**

This is a very difficult question for me as an avid reader who consumes many versions of the written word. In fact, my bucket list goal for this year is to read 100 books. “Death Be Not Proud” by John Gunther, which I found via a rummage sale when I was a teenager, became a literally guidepost toward medicine. It is instilled with humanity and reminders of how far medicine has come, yet how far we still must travel. “The Road Not Taken” by Robert Frost served as a north star reminding me that my unconventional career choices have indeed made all the difference. Finally, I read “The Alchemist” by Paulo Coelho almost every year and always find new insights.

